# Influence of temperature and pH on induction of Shiga toxin *Stx1a* in *Escherichia coli*

**DOI:** 10.3389/fmicb.2023.1181027

**Published:** 2023-07-06

**Authors:** Vinicius Silva Castro, Skyler Ngo, Kim Stanford

**Affiliations:** Department of Biological Sciences, University of Lethbridge, Lethbridge, AB, Canada

**Keywords:** Shiga-toxigenic *Escherichia coli*, enterohemorrhagic *Escherichia coli*, prophages, hydrogen-ion concentration, Shiga toxins

## Abstract

Shiga toxin-producing strains represent pathogenic group that is of concern in food production. The present study evaluated forty-eight *E. coli* isolates (11 with intact *stx* gene, while remaining isolates presented only *stx*-fragments) for Shiga toxin production. The four most expressive *stx*-producers (O26, O103, O145, and O157) were selected to evaluate effects of pH (3.5, 4.5, and 7) and temperature (35, 40, and 50°C). After determining acid stress effects in media on Stx-induction, we mimicked “*in natura*” conditions using milk, apple, and orange juices. Only isolates that showed the presence of intact *stx* gene (11/48) produced Shiga toxin. In addition, acid pH had a role in down-regulating the production of Shiga toxin, in both lactic acid and juices. In contrast, non-lethal heating (40°C), when in neutral pH and milk was a favorable environment to induce Shiga toxin. Lastly, two isolates (O26 and O103) showed a higher capacity to produce Shiga toxin and were included in a genomic cluster with other *E. coli* involved in worldwide foodborne outbreaks. The induction of this toxin when subjected to 40°C may represent a potential risk to the consumer, since the pathogenic effect of oral ingestion of Shiga toxin has already been proved in an animal model.

## 1. Introduction

*Escherichia coli* (*E. coli*) is important in clinical medicine (Harris et al., 2018), food production (Castro et al., 2017), animal production (Lavon et al., 2019), and biotechnology (Börner et al., 2019). *E. coli* has extreme genetic diversity and encompasses multiple serogroups and pathogenic groups (Aijuka et al., 2018), as well as non-pathogenic strains (generic *E. coli*; Lorenz et al., 2020). Among the pathogenic groups, the Shiga toxin-producing *E. coli* (STEC) represent an important group of pathogens, being related to food production and clinical medicine, due to their potential to cause foodborne illness (Castro et al., 2017). The ability to produce Shiga toxin originated from *Shigella dysenteriae* type 1 (Scheutz et al., 2012). The genetic origin of STEC helps to explain the evolutionary dynamism of *E. coli* since this bacterium acquired a pathogenic factor from *Shigella* in an originally enteropathogenic *E. coli* (EPEC; O55; Zhou et al., 2010).

Shiga toxins are based on the pentamer AB system where five equal B portions are linked to a toxic A unit (Sixma et al., 1993). Shiga toxins are encoded by *stx* genes originating from prophages integrated into the bacterial genome (Ohnishi et al., 2001). Shiga toxin-encoding prophages codify two types of toxins: *stx1* (*stx1a*, *stx1c*, and *stx1d*), or *stx2* [*stx2a*, *stx2b*, *stx2c*, *stx2d*, *stx2e*, *stx2f*, *stx2g,* and other new variants (Shen et al., 2022)]. For the Stx toxin to have activity, it is necessary that the bacterium binds to the host cell through adherence mechanisms, and then produce Shiga toxin which will internalize by a cellular invagination and reach the cytosol of the cell with subsequent damage during the protein translation stage (Castro et al., 2017). The production of Shiga toxin can be induced through external stressors that lead the *E. coli* cell to produce the toxin as a stress response (Fang et al., 2017). Among potential stressors, the use of UV-C, high temperatures, oxidizers, and the use of antibiotics have been previously addressed (Fang et al., 2017; Yang et al., 2018; Zhang et al., 2020). In addition, pathogenesis caused by oral ingestion of Shiga toxin has been determined in mice for both toxins (Stx1 and Stx2; Kurioka et al., 1998; Rasooly et al., 2010; Mohawk and O’Brien, 2011; Russo et al., 2014), being that the Stx2 toxin is potentially more lethal than Stx1 (Mohawk and O'Brien, 2011). However, although the effect in mice has been established, a similar mechanism is still unclear in humans. An important point is that although there is still no confirmation that the ingestion of Shiga toxin in food poses a risk to the consumer, the induction of these phages may indicate a greater predisposition to virulence by the *E. coli*. Recently Filipiak et al. (2020) revealed that the simple presence of the same *stx*-prophage in different isolates resulted in varied Shiga toxin induction, and the efficiency of induction seemed to be related not only to the type of *stx*-prophage but also to the inducing agent used and the host where bacteria were present. In addition, stimulation of *stx*-prophage can lead to dispersion of the phage in the microbial environment and the emergence of other strains with this virulence factor integrated in their genome (Martínez-Castillo and Muniesa, 2014). Furthermore, the presence of free *stx*-prophages can lead to incorrect detection of risks to food safety by causing false-positive PCR results (Joris et al., 2011; Martínez-Castillo and Muniesa, 2014; Castro et al., 2021).

Therefore, in the present study, we evaluated the response to two *stx*-prophage inducing factors (pH and temperature) in four STEC previously isolated (Stanford et al., 2016) and characterized genetically by whole genome sequencing (WGS; Castro et al., 2021). In this way, we tested a possible relation between the production of Shiga toxin and type of media (three liquid foods: milk, orange, and apple juices; all foods contain iron, a *stx1* phage regulator; Wagner et al., 2002). Finally, we investigated the genetic relatedness of our sequence data with that of other isolates obtained from animal reservoirs, the environment, and clinical disease cases through a phylogenetic tree, to understand and characterize the differences in *stx* phage induction.

## 2. Materials and methods

### 2.1. Culture samples

In the present study, we screened 48 isolates of *E. coli* previously characterized by WGS in the study of Carter et al. (2021). These isolates were initially studied due to variable results in the detection of *stx* gene (responsible for encoding the Shiga toxin) through PCR. All 48 isolates were evaluated for *in vitro* production of Shiga toxin, using a commercial kit (Ridascreen® Verotoxin, R-biopharm, Darmstadt, Germany). Therefore, we reactivated on MacConkey agar (Dalynn Biologicals, Calgary, Canada) isolates that were stored at −80°C, contained in Trypticase Soy Broth (TSB) with 10% glycerol. A colony from the MacConkey agar was then selected, inoculated in 9 mL of Luria-Bertani broth (LB; Fisher, USA), and incubated for 18 h at 35°C, with frequent agitation at 100 rpm in a shaker (VWR International, Bridgeport, USA). A 100 μL aliquot of the broth containing *E. coli* was collected and added to a new tube containing 4 mL of LB and 20 mM of EDTA (Sigma-Aldrich, Steinheim, Germany). This mixture was incubated for 2 h at 35°C with frequent agitation at 100 rpm. EDTA was used to replace antibiotics as Shiga-toxin promoters (Imamovic and Muniesa, 2012).

An aliquot of 100 μL of this combination of LB + EDTA was then centrifuged at 2,500 x *g* for 5 min and then included in a 96-well plate for assay of Shiga toxin. After sample insertion in the 96-well plate, all steps were followed according to the manufacturer’s protocol. Plate readings were performed on a plate spectrophotometer reader (Genesys 20 spectrophotometer, ThermoFisher Scientific, Waltham, USA), at a wavelength of 450 nm. After Shiga toxin expression was determined for the 48 isolates, we selected four to be tested in the temperature and pH treatments. Inclusion criteria were based on the maximum amount of toxin produced in the trial for each serogroup evaluated. Accordingly, we selected four isolates belonging to serogroups: O26 (CAP04), O103(CAP02), O145(CAP32), and O157(CAP10) for further analysis.

### 2.2. Temperature and pH

To investigate the effects of temperature and pH on the induction of *stx*-prophage, we initially used LB broth as a control, due to the presence of nutrients such as peptone and yeast extract. In addition, this broth had a pH of 6.9 ± 0.2, which contributed to choosing this medium as a negative control. To evaluate effects of pH, we used lactic acid (Sigma-Aldrich, Steinheim, Germany) as an acidifier to reach pH values of 3.5 ± 0.2 and 4.15 ± 0.3. Thus, the three pH ranges were approximately 3.5, 4.15, and 7. The second activation was performed using a new tube containing 4 mL of acidified LB (pH 3.5, 4,5), or a control group (Fresh LB pH 7). This second bacterial activation was incubated for 2 h at 35°C, with frequent agitation of 100 rpm in a shaker (VWR International, Bridgeport, USA). Subsequently, we applied temperature as a stress factor. Since pH is an intrinsic factor, we aimed to expose bacteria first to a range in pH (mimicking food) and then to temperature (mimicking heat treatment or storage). Therefore, we took 100 μL from the second bacterial activation (acidified and fresh LB) and included it in a microtube containing 900 μL of LB. The microtubes were exposed to temperatures of 35 (negative control), 40, and 50°C for 1 h. To reach this temperature we used a dry incubator with a shaker at 100 rpm. Subsequently, the microtubes were centrifuged at 2,500× *g* for 5 min and a 100 μL aliquot of the supernatant was collected and included in a 96-well plate. Finally, the reagents of the Shiga toxin measurement kit were included, and the steps were followed according to the manufacturer’s recommendations.

### 2.3. Milk and fruit juices to simulate stress on STEC

After the analysis using LB as media, we compared Shiga toxin expression values obtained in LB with liquid foods to assess the potential of bacteria to produce Shiga toxins in a “real world” condition, especially in cases where abuse of transport and storage temperature occurs. We used three foods purchased in grocery stores that had pH values close to those tested using LB broth and lactic acid. Therefore, we tested apple juice (pH 3.55 ± 0.4), orange juice (pH 4.12 ± 0.3), and milk containing 2% fat content (pH 6.8 ± 0.4). All juices and milk are pasteurized products and were tested on MacConkey agar for the presence of *E. coli* and were free of this bacterium. Thus, we performed a substitution of LB broth used in the second activation for these foods, respecting the same quantities inoculated and times of incubation. An important point was that in all treatments (LB + acid, and foods), the bacterial count was performed in MacConkey agar and bacterial inhibition was determined through the difference between the initial and final bacterial counts.

### 2.4. Quantification of Shiga toxin and cut-off values

The commercial kit Ridascreen® (Verotoxin, R-biopharm, Darmstadt, Germany) is based on the ELISA method to determine the production of Shiga toxin, through the binding of the toxin with specific antibodies, also described as a ‘sandwich’ binding method. Therefore, the kit has negative and positive controls that were used as a parameter for the quality of the assay. The cut-off values of the manufacturer (Optical Density (OD) of negative control +0.15) were used as a standard to determine whether Shiga toxin was produced. Also, samples that were less than 10% different from the cut-off value were re-tested. Furthermore, we measured all values in duplicate and if there was a difference greater than 10% between each replicate, the analysis was repeated. Finally, we used the Shiga toxin-production scale proposed by Lenzi et al. (2016) to determine whether the sample was weakly positive (1+, if it was OD > 0.1–0.5), moderate (2+, if it was OD > 0.5–1.0), or strongly positive (3+ if it was OD > 1.0 and < 2.0), or 4+ (OD >2.0) for Shiga toxin production.

### 2.5. Whole genome sequence analysis

The isolates used for the temperature and pH tests were previously sequenced and the draft genomes were presented by Castro et al. (2020). In addition, all sequenced isolates are available under BioProject number: PRJNA601484, and BioSamples: SAMN13870034, SAMN13870012, SAMN13870007, and SAMN13870005. The short reads were obtained using Illumina MiSeq platform, and specific details were described in Castro et al. (2021). To verify a possible relatedness to other *E. coli* involved in outbreaks in animal, environmental, or clinical cases, we selected 88 sequences using the NCBI tool entitled Pathogen Detection.^1^ Therefore, we use the search word “Outbreak” and filtered the sequences based on the organism group “*E. coli* and *Shigella*.” Afterward, we downloaded the 88 assemblies that were classified according to the applied filter and proceeded to the phylogenetic analysis among the isolates. All sequences accessed are described in Supplementary Table S1.

For phylogenetic analyses, we performed annotation of the genomes using Prokka (Prokka: rapid prokaryotic genome annotation; version 1.14.6; Seemann, 2014). Default parameters were used with a minimum contig size of 250 bp. After that, the genomes annotated in gff format were submitted to a core-genome alignment using Roary (version 3.13.0; Page et al., 2015), with a minimum percentage identity for blastp of 95 and 10% of isolates containing the gene that must be into the core-genome. After that, the core-genome alignment obtained by Roary was applied to IQ-Tree (version 2.1.2; Nguyen et al., 2014) to build an evolutionary tree with construction from multiple sequences. The specified number of bootstrap replicates was 1,000. Later the phylogenetic tree obtained was annotated using iTol (Interactive tree of life; version 6.5.8), and Microsoft PowerPoint (version 365; Microsoft 2022).

### 2.6. Statistical analysis

Data were compared using ANOVA with Tukey as a posthoc test to compare mean values at a 5% of the significance level. All experiment were conducted three times (three independent replicates) and in each time, two replicates were performed. In addition, all analyses were conducted using Minitab version 19.1 software.

## 3. Results and discussion

### 3.1. STEC reduction and Shiga toxin production using “*in vitro*” tests for pH

**Figure 1:**
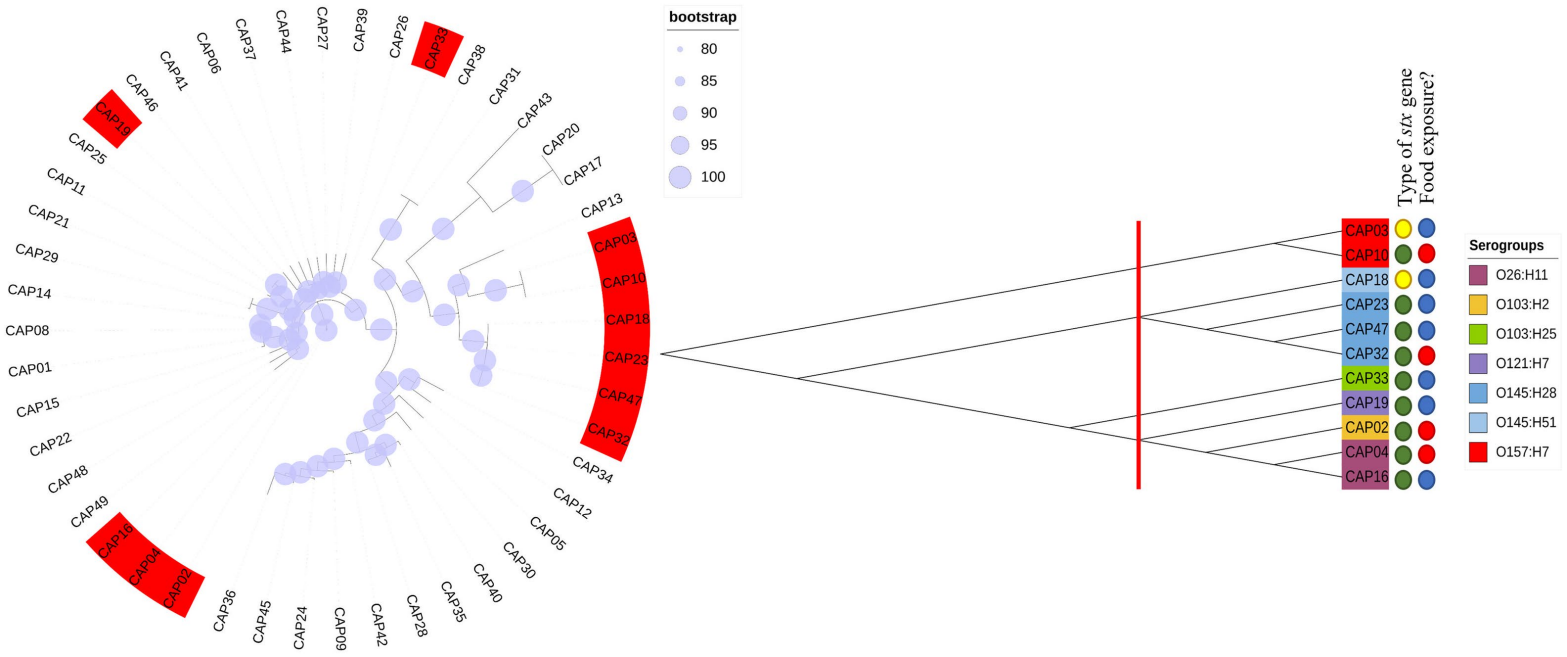
Phylogenetic trees using our 48 *E. coli* including 11 STEC isolates. We performed a first phylogenetic tree with all isolates tested in the first trial. In red, are isolates that were positive for Shiga toxin production. Later we performed a new phylogenetic tree with only the 11 Stx producers. Thus, we checked 3 different groups and selected the isolates for further evaluation according to their branch of the phylogenetic tree.

All 48 *E. coli* isolates analyzed by Castro et al. (2021) were tested for Shiga toxin expression in the present study. Thus, all that showed the presence of the intact *stx* gene (11/48) produced Shiga toxin, and all isolates which were *stx*-negative by WGS, but that presented fragments of the *stx* gene were not capable of producing Shiga toxin “*in vitro*” (Figure 1). In this way, we selected four isolates to be tested at different pH and temperature, with the criteria adopted based on the serogroup, the amount of toxin production based on the first trial (Figure 1) and their presence in each phylogenetic tree branch.

**Figure 2:**
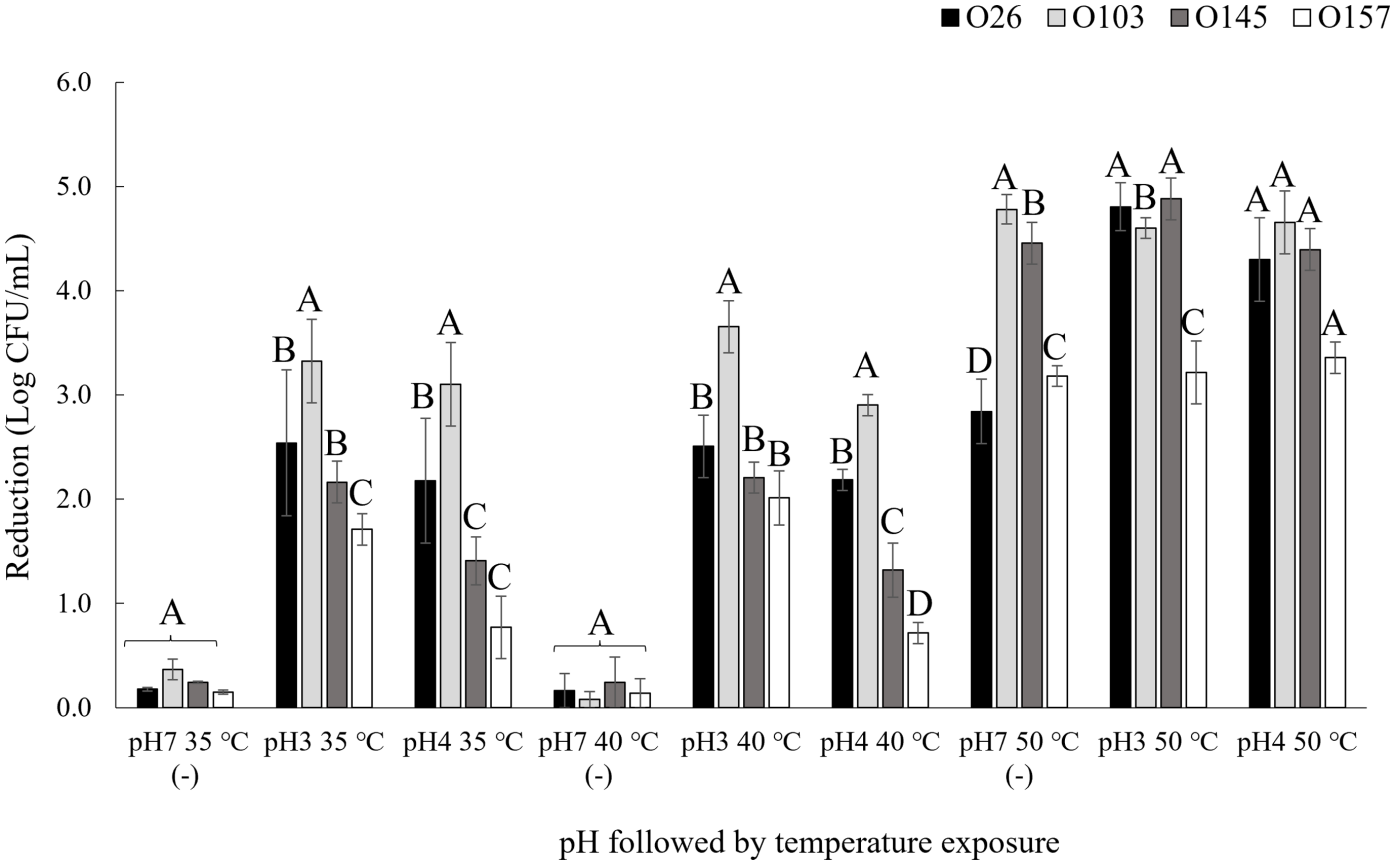
Bacterial reduction after pH and thermal exposure. Isolates were submitted to pH and temperature incubations of 1 h each. pH 7 (−) was used as negative control. Bacterial reduction was calculated using the control population for each isolate before treatment exposure minus the population after exposure to the pH and temperature treatments. Different capital letters indicate a significant difference between isolates (*p <* 0.05) comparing into each treatment using Tukey as *post hoc* means test.

Four different serogroups were tested at three different pH values (3.5, 4.15, and 7) and at three different temperatures: 35 (control), 40, and 50°C. Bacterial load reduction after exposure to pH and temperature are shown in Figure 2. Based on the results obtained, it was possible to verify that the isolates showed different profiles of bacterial inhibition in each treatment tested, such as the serogroup O26, which showed the lowest reduction when incubated in pH 7 at 50°C (*p <* 0.05) but was one of the serogroups with the greatest reduction when incubated at 50°C at a pH 3.5 or pH 4.15 (Figure 2). The important point is that *E. coli* differs from other bacteria by having different mechanisms of resistance to acidic pH, such as the presence of decarboxylation systems of glutamate or arginine (Foster, 2004), and the presence of resistance genes that help to regulate stress responses such as *rpoS* (Schellhorn, 2020). Thus, genetic diversity may explain differences in acid stress resistance profiles. In this way, the survival of each serogroup may be affected differently by pH and combining low pH with temperature may further enhance microbial reduction (Blackburn et al., 1997). A point to highlight was that in the present study we used lactic acid as an acidulant since this acid is naturally present in a diverse number of foods, mainly in fermented products (Kandasamy et al., 2018).

**Figure 3:**
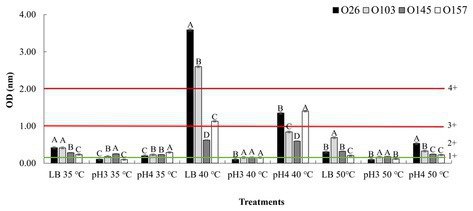
*Stx* expression after pH and temperature exposure. Shiga toxin production was calculated based on optical density (OD) values. Positive and negative kit controls were used to ensure test quality and to calculate negative or positive sample breakpoints for Shiga-toxin production. The cut-off values of the manufacturer for Shiga toxin producing positive are OD of negative control +0.15. Differences were calculated between serogroups in each treatment. Different letters indicate a significant difference (*p <* 0.05) based on *post hoc* test (Tukey means test). Toxin classifications followed criteria established by Lenzi et al. (2016): 1+ (weak positive), 2+ (moderate), 3 and 4+ (strong positive).

To approach the data, we used the classification used by Lenzi et al. (2016), in which the absorbance values were divided into ranges from 1+ (weak toxin formation) to 4+ (strong toxin formation), which represent the amount of toxin produced (Figure 3). In the present study, we used the same Shiga toxin measurement kit as the study by Lenzi et al. (2016), and in all cases we subtracted the value of the OD (negative control) from the OD (sample). It is important to note that in a study performed by Parma et al. (2012), the authors found that all Stx1 toxin that crossed the +1 threshold of the Ridascreen kit were also positive by the Vero cell toxicity assay. The amount of Shiga toxin is especially important in the case of Stx1 toxins because they are less potent than Stx2 (Kurioka et al., 1998). In the study performed by Tesh et al. (1993), the authors determined the 50% lethal dose of Stx2 to be 400 times lower than that of Stx1. Thus, it was necessary to include 400x more Stx1 than Stx2 to induce the lethal dose in 50% of populations of mice.

As shown in Figure 3, an important point was that in all samples with acidic pH 4 and neutral pH 7, at the temperature of 40°C there was a higher toxin production overall than that at 35°C. However, when analyzing the behaviors within the same temperature range, the use of pure LB (pH ~ 7) allowed a greater induction of Shiga toxin when compared to treatments using acid pH (Figure 3). The data revealed that the closer the culture medium is to pH neutrality, the greater is the action of temperature in inducing the prophage. However, when comparing the effects of temperature in pure LB, we note that only in the non-lethal heating was there a high induction of Shiga toxin.

Our results corroborate other studies that evaluated the effects of cellular stress on Shiga toxin induction. In a study performed by Carey et al. (2008), the authors also showed that low pH has the effect of down-regulating the expression of Shiga toxin, and at closer to neutrality, a greater expression of this gene was observed. Furthermore, an interesting point was that in the study performed by Fang et al. (2017) the authors highlighted that a temperature of 50°C did not influence the induction of *stx2*-prophage. Our results corroborate the data tested at the same temperature of 50°C when compared to the LB control group at 35°C. However, when we applied a non-lethal temperature of 40°C there was an over-expression *stx1* by two serogroups (O26 and O103). We hypothesize that at temperatures where there is bacterial death, the cell would express several resistance and metabolic genes that would make over-expression of *stx* difficult. Thus, at non-lethal temperature, an up-regulated expression of prophage *stx* could be associated with a cellular response as a reaction to the stress experienced. In this way, it is worth mentioning that in situations of microbial or viral infection, the human body tends to cause an increase in body temperature (fever) (Blatteis, 2010). Thus, this over-expression may have an evolutionary relationship of adaptation to the immune response of the animal host, which increases body temperature to seek to control the infection. Further studies may answer whether this small variation in body temperature (36–40°C) may have a correlation with the production of Shiga toxin. In addition, in food production environments the use of heating is common (Pereira and Vicente, 2010), and this heat could contribute to the induction of this toxin in the food. Therefore, we also aimed to investigate the effects of pH and temperature treatments using different liquid foods based on their intrinsic pH values.

### 3.2. Use of apple, orange juices, and milk at 2% of fat content for stx production

**Figure 4:**
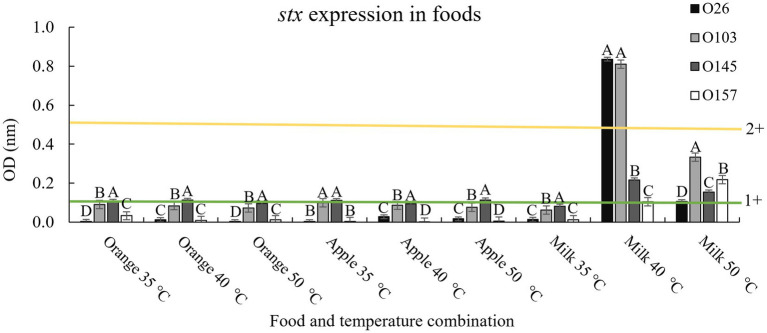
*Stx* expression in food and after thermal exposure. Shiga toxin production was calculated based on optical density (OD) values. Positive and negative kit controls were used to ensure test quality and to calculate negative or positive sample breakpoints for Shiga-toxin production. The cut-off values of the manufacturer for Shiga toxin producing positive are OD of negative control +0.15. Differences were calculated between serogroups in each treatment. Different letters indicate a significant difference (*p <* 0.05) based on *post hoc* test (Tukey means test). Toxin classifications followed criteria established by Lenzi et al. (2016): 1+ (weak positive), 2+ (moderate).

To determine possible effects of pH on the matrix, we used three liquid foods to verify the effect obtained in LB. For pH 3.5 we used a 100% undiluted apple juice (pH 3.3 ± 0.2), for pH 4.15 we used 100% orange juice (pH 4.1 ± 0.3), and for pH 7 we used cow’s milk with 2% fat content (pH 6.9 ± 0.2). The results are shown in Figure 4.

In general, the results followed the same pattern presented when using only LB and lactic acid, with the temperature of 40°C having greater Shiga toxin expression when applied in a medium with a pH closer to neutrality (milk; Figure 4). However, it is worth noting that although the Shiga toxin production pattern indicated that a pH closer to neutral combined with a non-lethal temperature of 40°C induced the prophage to produce more toxin, the amount of toxin produced was below values obtained in LB at 40°C (4+ vs. 2+, Figures 3, 4). The three foods used contain trace amounts of iron, which even in low concentrations is a strong regulator of Stx1 (Wagner et al., 2002). Therefore, it was expected that iron and other compounds intrinsic to foods could interact with the bacteria and prevent the maximum potential of producing Shiga toxin. Similarly, milk fat globules have been shown to prevent STEC cell adhesion to epithelial cells (Bagel and Sergentet, 2022). The study by Bagel and Sergentet (2022) represents only one milk component that could interact with STEC, which demonstrates the complexity involved when evaluating toxin production using food as a medium.

The *E. coli* bacterium has several mechanisms to protect itself from adverse pH. One of our hypotheses focused on the production of outer membrane vesicles. These membranes are based on the dissociation of the outer membrane into specific zones (Mozaheb and Mingeot-Leclercq, 2020). Under adverse conditions, these vesicles could guarantee the maintenance of membrane integrity. However, as a disadvantage, there could be a delay in the phenotypic resistance response (Mozaheb and Mingeot-Leclercq, 2020). Thus, the formation of a vesicle to adapt to the acidic environment might preclude the production of Shiga toxin as a defense mechanism. In contrast, when a non-lethal temperature was used, the cell did not need to focus on survival and was more able to produce Shiga toxin. In addition, another factor that makes bacteria resist thermal stress is the presence of the *degP* gene. In a study by McBroom and Kuehn (2007), the authors highlighted the versatility of this gene which could act both as a protease and as a chaperone, with an impact on combating protein misfolding in the periplasm. In the present study, all 4 strains used in the assays with temperature and at different pH had intact *degP* genes in their genome. Consequently, these strains indicate a possibility of adaptation to heat which may have enhanced their production of Shiga toxin.

In addition, other studies corroborate the impact of temperatures above the optimal growth point (35°C), but below the onset of cell lethality on the induction of *stx* prophages (Łoś et al., 2009). In the study performed by Zhang et al. (2020), the results were the opposite of those of the present study, with O145 and O157 (both *stx2* positive) being higher producers of Shiga toxin than O26 and O103 (both *stx1* positive) when exposed to various stressors. It is important to note that Stx2 is normally much more cytotoxic than Stx1 (up to 1,000 times more cytotoxic; Bertin et al., 2001). Taking these results into account, the presence of a *stx2* gene is likely to cause more infection in humans than *stx1*. So, more important than the bacterial serogroup is the type of toxin present in each isolate. In the present study, all isolates presented *stx1* (toxin variant a; Castro et al., 2021). This variant has a historical association with bovine isolates (Boerlin et al., 1999) and has been common in cases of human infections in Switzerland (Fierz et al., 2017). In addition, serogroups O26 and O103 presented prophage BP-4795 (NC_004813). Serogroup O145 presented prophage PA28 (NC_041935), and serogroup O157 had prophage *stx1* converting phage (NC_004913) (Castro et al., 2021). It is worth mentioning that in the present study, both isolates that showed the presence of prophage BP-4795 (O26 and O103) were those related to the highest amount of toxin produced after induction at 40°C. However, even though the two isolates had the same prophage, there was a significant difference (*p <* 0.05) in the amount of toxin produced between them with different temperatures and media (Figures 3, 4). In a study performed by Filipiak et al. (2020) the authors pointed out the type of phage present, the bacterial genome, and the inducing agent influenced the production of Shiga toxin. Therefore, our study corroborates that the type of *stx* prophage present in the cell, the presence of other virulence factors, and stress resistance may have a greater influence on the toxin production capacity than the *E. coli* serogroup alone. Consequently, we evaluated the genetic profiles of the isolates used in the present study to identify possible differences that could explain the variations between the quantities of toxins produced during cellular stress.

### 3.3. Possible genes responsible for stx gene overexpression under sub-lethal temperature and lack of acid resistance genes

**Figure 5:**
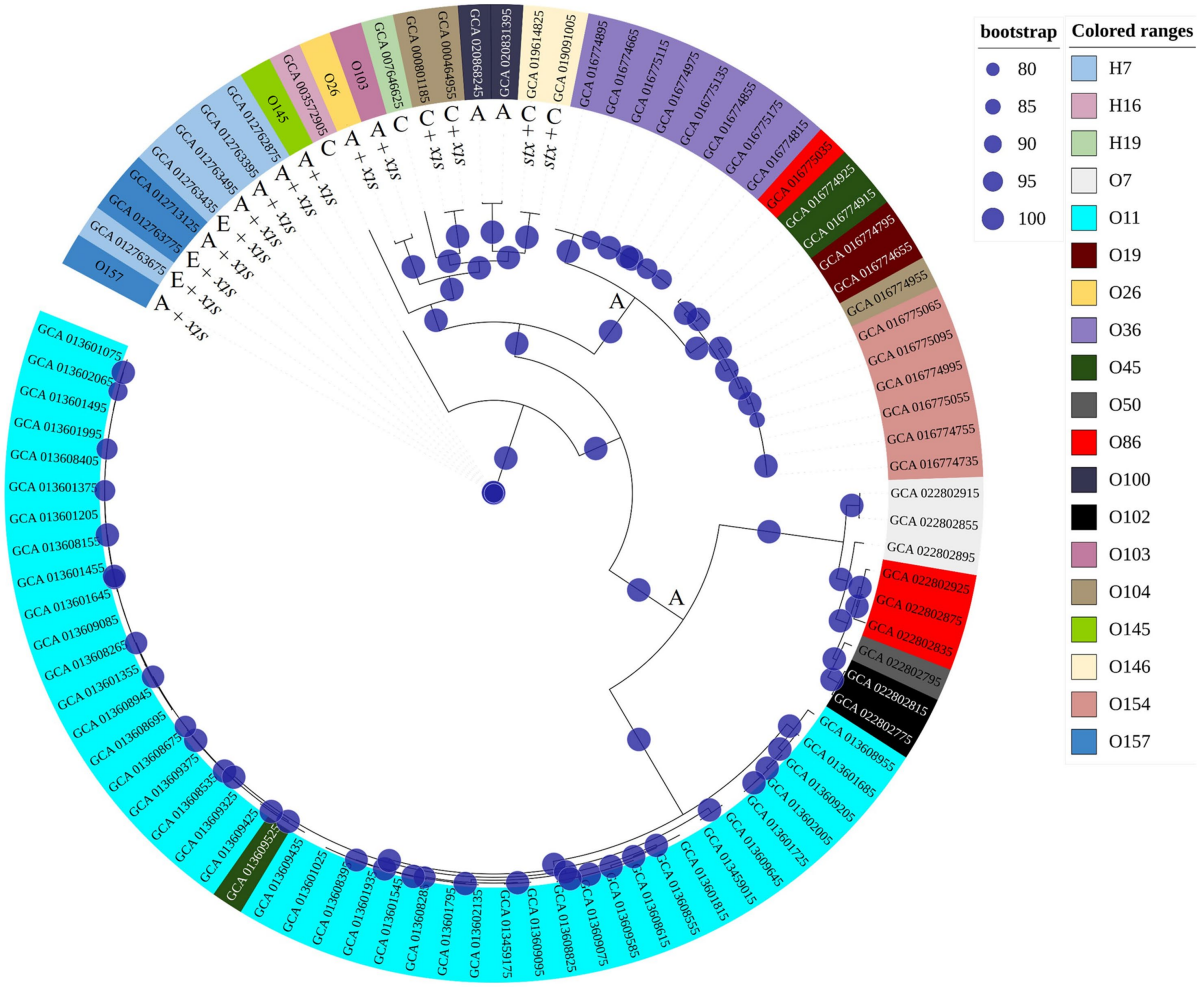
Phylogenetic tree using genomes tested in this study and other sequences involved in environmental, clinical, or animal isolation. The isolates came from multiple sources. A: Animal host; E: environmental isolation; C: Clinical cases or outbreaks. Minimum values of 80 bootstraps were selected to support differences between genomes. *E. coli* labelled as O26, O103, O145, and O157 represent isolates tested for pH and heat exposure in the present study.

Considering impacts of pH and temperature, isolates from serogroups O26 and O103 showed the highest expression of Shiga toxin, both in LB and milk at 40°C. In this sense, we investigated the possible similarities and differences between genomes that could explain this heightened production of Shiga toxin. We compared the four tested isolates with another 88 obtained through Pathogen Detection (NCBI). Thus, we found that the core-genome of O26 and O103 isolates were associated with a cluster that mostly involves clinical cases (Figure 5). For example, except for GCA_020868245 and GCA_020831395 which were also isolated from animals, all others were related to human cases or pathogenic outbreaks (Figure 5). Thus, the Shiga toxin expression values of the O26 and O103 isolates can be related to their infectious capacity since these two isolates that presented the highest amount of Shiga toxin when subjected to heat stress were also related to a cluster involved in clinical cases.

These results corroborate the study performed by Carter et al. (2021) where the authors identified that an isolate from a bovine reservoir was related to other isolates from human cases. However, in contrast to the study by Carter et al. (2021), the present study found STEC with stx1 related to clinical isolates, which leads us to suppose that stx2a-prophages may not be the only source of hypervirulent STEC. It is worth mentioning that the isolates were selected from NCBI based only on the species (*E. coli* and Shigella), without any pathogenic group having been previously selected. Thus, among the 92 genomes used for constructing the phylogenetic tree, 88 were retrieved from NCBI, and 4 isolates (O26, O103, O145, and O157) used in the present study were included to assess the genetic relationship with the other isolates. The results obtained indicate the presence of 3 clusters (Figure 5), the first isolated from animals and the environment (O145, and O157 isolates used in the present study were present in this cluster). The second cluster presented strains of human clinical and animal origin (including the O26 and O103 of the present study), and the third and last cluster presented only animal strains. Thus, among the 92 isolates analyzed, 15 were positive for Shiga toxin (Figure 5). It is worth mentioning that the isolates that produced the most Shiga toxin in juices (O26 and O103) were included in the clinical cluster. In this sense, among the clinical cluster, 40% (6/15) presented the stx1 gene (two stx1a (O26, and O103); two stx1c (GCA_019091005.1, GCA_019614825.1); and two stx2a genes (GCA_000464955.2, GCA_000801185.2)). Consequently, the presence of the stx gene represents one of the many virulence factors found in *E. coli*, and due to the gene transfer capacity of bacteria, many virulence factors can be shared between different pathogenic groups, in addition to the presence of strains with hybrid virulence factors (Paletta et al., 2020). Among the *E. coli* present in the cluster with clinical infections, we can highlight strain GCA_007646625 which represents an *E. coli* classified as an atypical EPEC isolated in a case of human diarrhea (GCA_007646625 – NCBI). STEC share several genetic components with EPEC due to their evolutionary origin being derived from an EPEC O55 (Koudelka et al., 2018). Factors linked to virulence capacity, cell adhesion, and serogroup homology between the EPEC and STEC groups were addressed by Spears et al. (2006).

From the results obtained in the production of Shiga toxin and the relationship between the major toxin producers and other the cluster of *E. coli* involved in clinical cases of infection, we sought to evaluate the differences between the genes present in isolates O26 and O103 (greater production of Shiga toxin) in relation to isolates O145 and O157 (lower production of Shiga toxin with the stresses tested). The presence of particular genes of interest were evaluated (Table 1).

**Table 1:** Genes present in O26 and O103 (higher stx-prophage inducers) which were absent in O145 and O157 (less stx-prophage inducers).

Gene	Functions	References
*ElfA*	*ElfA* is an accessory colonization factor that contributes to the adherence of bacteria to human intestinal epithelial cells and to animal intestinal tissue *in vitro*	Samadder et al. (2009)
Chaperone protein DnaJ	Participates actively in the response to hyperosmotic and heat shock by preventing the aggregation of stress-denatured proteins and by disaggregating proteins. Unfolded proteins bind initially to DnaJ; upon interaction with the DnaJ-bound protein, DnaK hydrolyzes its bound ATP, resulting in the formation of a stable complex.	Siegenthaler et al. (2004), Zzaman et al. (2004)
Intimin	Multifunctional protein is required for efficient pedestal formation in host epithelial cells during infection. The extracellular region acts as a receptor for bacterial intimin, allowing the bacterium to attach tightly to the host-cell surface.	DeVinney et al. (1999)
*ompD*	ompD is a porin that influences cell permeability. The abundance of OmpD decreases in response to low pH in *Salmonella.*	Santiviago et al. (2003)
*eaeB*	Required for the intimate attachment of *E.coli* to the epithelial cells.	Donnenberg et al. (1993)
*fimA*	Fimbriae (also called pili), polar filaments radiating from the surface of the bacterium to a length of 0.5–1.5 micrometers and numbering 100–300 per cell, enable bacteria to colonize the epithelium of specific host organs.	Boudeau et al. (2001)

The genes present here are likely related to differences in induction results among isolates. Several other genes were present only in O26 and O103 in contrast to O145 and O157. All gene presence/absence were included in Supplementary Table S2.

In general, we found that important genes related to cell adhesion and stress tolerance were present in the two isolates with greater Shiga toxin induction compared to serogroups O145 and O157 (Table 1). Based on the data obtained in Table 1, we highlighted the presence of *ompD* gene. This gene is a cellular component involved in the cell outer membrane (Liu et al., 2017). In addition, the *ompD* gene also acts as a porin and can act on the transmembrane ion transport (Li et al., 2019). Furthermore, some studies have indicated that negative regulation of this gene leads to a decrease in cell membrane permeability (He and Ahn, 2011; Zou et al., 2012). Cell permeability has the function of protecting the bacteria against damage from harmful components (Xiang et al., 2019). In studies that used mild temperature, an increase in cell permeability was observed (Xiang et al., 2019) Therefore, the presence of the *ompD* gene could explain the greater survival of s O26 and O103 isolates at pH 7 at 40°C (Figure 3), and this survivability can be related to Shiga toxin production when we exposed O26 and O103 to milk at 40°C (Figure 4). However, future studies using RNA sequencing must be conducted to determine the real impact of *ompD* gene in relation to this higher isolate survivability.

The presence of these genes in O26 and O103 isolates may explain their clustering with others involved in clinical cases. It is noteworthy that cell adhesion between bacteria and the host is a key step for the Shiga toxin to be internalized and cause damage to the host (Castro et al., 2017; Paletta et al., 2020). However, the injury that oral ingestion of preformed toxin can cause in humans is still unclear. In this sense, Rasooly et al. (2010) investigated the impacts of administering Shiga toxin directly into the stomach of mice and noted that a portion of the toxin reached organs and caused damage. Furthermore, the authors emphasized the toxin’s ability to survive even at pasteurization temperatures (Rasooly et al., 2010). Russo et al. (2014) found that both routes of intoxication (oral versus intraperitoneal) elicited kidney damage, weight loss in the animal, and electrolyte imbalances., Even with the hypothesis of a preformed toxin in the food not causing damage after ingestion, other negative outcomes could arise when the bacterium undergoes stress and triggers a metabolic response (dos Santos Rosario et al., 2021). Among negative outcomes, based on the present study, we can mention the possible additional risk of STEC that presented high production of Shiga toxin and a phylogenetic relationship with other clinical isolates. Thus, the increased induction of prophage *stx* under stresses such as high temperatures leads us to assume that these STEC would also be more able to cause infection in people consuming the contaminated food.

Furthermore, little is known about the impacts of *stx* gene prophage induction with a possible increase in prophage dispersion within the bacterial community. In a study performed by Martínez-Castillo and Muniesa (2014), the authors addressed the impacts that free *stx*-phages could have when dispersed. The main problem would be the emergence of new virulent strains, as well as interference in the detection of positive samples in food safety applications (Brown-Jaque et al., 2016). In addition, several studies have reported that bacteria previously exposed to stressors increased their survival and potential pathogenicity (Mutz et al., 2020). A study performed by Kim et al. (2016) evaluated survival in a synthetic gastric fluid (SGF) after pre-adaptation of STEC strains in pineapple juice. The results indicated that after previous exposure to stress caused by the acidity of the juice, the isolates showed up-regulation in genes related to acidity resistance, such as the SGF environment (Kim et al., 2016). Furthermore, in a study performed by Castro et al. (2019), the authors demonstrated that isolates exposed to heat stress and UV-C light had a better ability to survive when exposed to SGF. Thus, the previous exposure of *E. coli* isolates to cellular stress seems to reinforce cell resistance in a future hostile environment. In this sense, a heightened expression of Shiga toxin in food leads us to suppose that if the bacterium overcomes the natural barriers of the human body and manages to establish in the intestinal microvilli, there may be an aggressive infectious response, motivated by pre-adaptation to stress. New studies may shed light on this possible factor of virulence potentiation through previous stress.

## 4. Conclusion

The results of the present study corroborate other findings that demonstrate the decrease in the production of Shiga toxin at lethal heating temperatures (50°C), and low pH values (3.5 and 4.15). However, at non-lethal heating temperatures (40°C), an overexpression of Shiga toxin was detected at neutral pH (LB or Milk ~ pH 7). Also, two isolates of serogroups O26 and O103 showed higher Shiga toxin expression than those of serogroups O145 and O157 at a temperature of 40°C. In this sense, when comparing the isolates used in the present study with other *E. coli* obtained from the NCBI database (environmental and clinical strains), the two serogroups with the highest production of Shiga toxin were present in a cluster with related to human infections.

In addition, we noticed that isolates of serogroups O26 and O103 had a greater diversity of genes involved in host cell adhesion in contrast to those of serogroups O145 and O157. So, our study emphasizes that STEC with greater pathogenic potential may produce more Shiga toxin in food, and although oral pathogenesis is still unclear in humans, the possibility of this infection has been demonstrated in an animal model.

## Data availability statement

The datasets presented in this study can be found in online repositories. The names of the repository/repositories and accession number(s) can be found in the article/Supplementary material.

## Author contributions

VC and KS: conceptualization. VC: methodology, software, data curation, and writing—original draft preparation. VC and SN: formal analysis and investigation. KS: resources, writing—review and editing, supervision, project administration, and funding acquisition. All authors have read and agreed to the published version of the manuscript.

## Funding

This work was supported by Results-Driven Agricultural Research (RDAR) project #2021R014R and Beef Cattle Research Council (BCRC) project FOS.01.17.

## Conflict of interest

The authors declare that the research was conducted in the absence of any commercial or financial relationships that could be construed as a potential conflict of interest.

## Publisher’s note

All claims expressed in this article are solely those of the authors and do not necessarily represent those of their affiliated organizations, or those of the publisher, the editors and the reviewers. Any product that may be evaluated in this article, or claim that may be made by its manufacturer, is not guaranteed or endorsed by the publisher.

## Footnotes

^1^ https://www.ncbi.nlm.nih.gov/pathogens/
