# Supplementary material for: Influence of temperature and pH on induction of Shiga toxin Stx1a in Escherichia coli
Source: Front Microbiol. 2023 Jul 6;14:1181027. doi: 10.3389/fmicb.2023.1181027 (PMC10359099; doi:10.3389/fmicb.2023.1181027)
Supplement: Supplementary file 1 [file Data_Sheet_1.PDF]

Supplementary material. Gene presence in *E. coli* O26 and O103 strains and absence in O145 and O157 strains.

Gene	Annotation	No. sequences	Order within Fragment	Accessory Fragment	Min group size nuc.	Max group size nuc.	CAP2 – O103	CAP4 – O26	CAP10 – O157	CAP32 – O145
adhB_2	Alcohol dehydrogenase 2	2	1022	303	1148	1148	AJPELHOP_03668	IJBEAHBO_03838		
agaC_3	N-acetylgalactosamine permease IIC component 1	2	116	59	749	749	AJPELHOP_01728	IJBEAHBO_01720		
aglB_1	6-phospho-alpha-glucosidase	2	1630	362	638	638	AJPELHOP_03858	IJBEAHBO_02346		
ampG_2	Anhydromuropeptide permease	2	2456	28	1280	1280	AJPELHOP_03473	IJBEAHBO_03587		
appX	Putative cytochrome bd-II ubiquinol oxidase subunit AppX	2	418	847	92	92	AJPELHOP_04684	IJBEAHBO_04081		
axe1-6A	Carbohydrate acetyl esterase/feruloyl esterase	2	1599	49	1169	1169	AJPELHOP_03144	IJBEAHBO_02310		
azoR_1	FMN-dependent NADH-azoreductase	2	57	180	590	590	AJPELHOP_01892	IJBEAHBO_01884		
bglA_2	Aryl-phospho-beta-D-glucosidase BglA	2	62	60	1439	1439	AJPELHOP_03782	IJBEAHBO_04016		
bglB	6-phospho-beta-glucosidase BglB	2	1597	49	1394	1394	AJPELHOP_03142	IJBEAHBO_02308		
bglF_2	PTS system beta-glucoside-specific EIIBCA component	2	1596	49	1877	1877	AJPELHOP_03141	IJBEAHBO_02307		
bglG	Cryptic beta-glucoside bgl operon antiterminator	2	1595	49	836	836	AJPELHOP_03140	IJBEAHBO_02306		
bglH_2	Cryptic outer membrane porin BglH	2	1598	49	1616	1616	AJPELHOP_03143	IJBEAHBO_02309		
bglH_3	Cryptic outer membrane porin BglH	2	61	60	1673	1673	AJPELHOP_03783	IJBEAHBO_04015		
btuD_4	Iron import ATP-binding/permease protein IrtA	2	2454	28	1712	1802	AJPELHOP_03471	IJBEAHBO_03585		
cbl_2	HTH-type transcriptional regulator cbl	2	2437	318	305	305	AJPELHOP_03453	IJBEAHBO_03567		
cbrB	Inner membrane protein CbrB	2	1602	49	473	473	AJPELHOP_03147	IJBEAHBO_02313		
cbtA_1	Cytoskeleton-binding toxin CbtA	2	704	1	374	374	AJPELHOP_00714	IJBEAHBO_00674		
celA	PTS system cellobiose-specific EIIB component	2	66	60	311	311	AJPELHOP_03786	IJBEAHBO_04012		
cfaB	CFA/I fimbrial subunit B	2	1915	112	500	500	AJPELHOP_00102	IJBEAHBO_00741		
cfaE	CFA/I fimbrial subunit E	2	1917	112	926	926	AJPELHOP_00100	IJBEAHBO_00743		
chbA_2	PTS system N,N'-diacetylchitobiose-specific EIIA component	2	64	60	311	311	AJPELHOP_03784	IJBEAHBO_04014		
chbC_2	PTS system N,N'-diacetylchitobiose-specific EIIC component	2	65	60	1322	1322	AJPELHOP_03785	IJBEAHBO_04013		
cirA_2	Colicin I receptor	2	5011	159	992	992	AJPELHOP_04099	IJBEAHBO_04352		
cirA_3	Colicin I receptor	2	1738	124	992	992	AJPELHOP_05071	IJBEAHBO_05298		
cmoM_2	tRNA 5-carboxymethoxyuridine methyltransferase	2	2451	28	9482	9482	AJPELHOP_03468	IJBEAHBO_03582		
cycB	Cyclodextrin-binding protein	2	2194	32	1232	1232	AJPELHOP_04357	IJBEAHBO_02237		
dapH_2	2,3,4,5-tetrahydropyridine-2,6-dicarboxylate N-acetyltransferase	2	95	317	809	809	AJPELHOP_04517	IJBEAHBO_04638		
dctM_2	C4-dicarboxylate TRAP transporter large permease protein DctM	2	92	132	1301	1301	AJPELHOP_01704	IJBEAHBO_01696		
dgaE	D-glucosaminate-6-phosphate ammonia lyase	2	118	59	1109	1109	AJPELHOP_01730	IJBEAHBO_01722		
dgaF	2-dehydro-3-deoxy-phosphogluconate aldolase	2	119	59	743	743	AJPELHOP_01731	IJBEAHBO_01723		
dgaR	Transcriptional regulatory protein DagR	2	113	59	2840	2840	AJPELHOP_01725	IJBEAHBO_01717		
dgoA	2-dehydro-3-deoxy-6-phosphogalactonate aldolase	2	1537	71	617	617	AJPELHOP_03869	IJBEAHBO_02335		
dgoD	D-galactonate dehydratase	2	1536	71	1148	1148	AJPELHOP_03868	IJBEAHBO_02336		
dgoK1	putative 2-dehydro-3-deoxygalactonokinase DgoK1	2	1538	71	878	878	AJPELHOP_03870	IJBEAHBO_02334		
dgoT	D-galactonate transporter	2	1535	71	1292	1292	AJPELHOP_03867	IJBEAHBO_02337		
dhbE	2,3-dihydroxybenzoate-AMP ligase	2	2448	28	1565	1565	AJPELHOP_03465	IJBEAHBO_03579		
dlgD	2,3-diketo-L-gulonate reductase	2	1431	43	998	998	AJPELHOP_03380	IJBEAHBO_03542		
dltA	D-alanine--D-alanyl carrier protein ligase	2	2452	28	6107	6107	AJPELHOP_03469	IJBEAHBO_03583		
dnaB_3	Replicative DNA helicase	2	6	24	1376	1376	AJPELHOP_04838	IJBEAHBO_05006		
eae	Intimin	2	2093	365	2819	2819	AJPELHOP_03936	IJBEAHBO_04657		
eaeB	Protein EaeB	2	2092	109	944	944	AJPELHOP_03941	IJBEAHBO_04652		
elfA	Laminin-binding fimbrial subunit ElfA	2	2526	364	542	542	AJPELHOP_03715	IJBEAHBO_03907		
elfG_1	putative fimbrial-like protein ElfG	2	1575	100	1073	1073	AJPELHOP_03134	IJBEAHBO_02300		
eptC_2	Phosphoethanolamine transferase EptC	2	2037	11	1742	1742	AJPELHOP_04411	IJBEAHBO_04527		
espF(U)_1	Secreted effector protein EspF(U)	2	2133	34	623	623	AJPELHOP_03945	IJBEAHBO_04737		
espF(U)_2	Secreted effector protein EspF(U)	2	383	81	611	611	AJPELHOP_04673	IJBEAHBO_05386		
fimA_1	Type-1 fimbrial protein, A chain	2	735	399	548	548	AJPELHOP_00703	IJBEAHBO_00663		
flgB_1	Flagellar basal body rod protein FlgB	2	4157	7	344	344	AJPELHOP_03313	IJBEAHBO_03107		
flgC_1	Flagellar basal-body rod protein FlgC	2	4137	7	431	431	AJPELHOP_03312	IJBEAHBO_03106		
flgE_2	Flagellar hook protein FlgE	2	4139	7	1202	1202	AJPELHOP_03310	IJBEAHBO_03104		
flgF_2	Flagellar basal-body rod protein FlgF	2	4140	7	737	737	AJPELHOP_03309	IJBEAHBO_03103		
flgG_2	Flagellar basal-body rod protein FlgG	2	4141	7	785	785	AJPELHOP_03308	IJBEAHBO_03102		
flgH_1	Flagellar L-ring protein	2	4142	7	740	740	AJPELHOP_03307	IJBEAHBO_03101		
flgI_2	Flagellar P-ring protein	2	4143	7	1100	1100	AJPELHOP_03306	IJBEAHBO_03100		
flgL_1	Flagellar hook-associated protein 3	2	4146	7	929	929	AJPELHOP_03303	IJBEAHBO_03097		
flhB_2	Flagellar biosynthetic protein FlhB	2	4176	7	1139	1139	AJPELHOP_03332	IJBEAHBO_03126		
fliC	Flagellin	2	5101	193	1463	1463	AJPELHOP_01529	IJBEAHBO_03792		
fliC1	Flagellin 1	2	4149	7	914	914	AJPELHOP_03300	IJBEAHBO_03094		
fliD_1	Flagellar hook-associated protein 2	2	4150	7	1316	1316	AJPELHOP_03299	IJBEAHBO_03093		
fliD_2	Flagellar hook-associated protein 2	2	5100	193	1412	1412	AJPELHOP_01530	IJBEAHBO_03793		
fliE_2	Flagellar hook-basal body complex protein FliE	2	4169	7	341	341	AJPELHOP_03325	IJBEAHBO_03119		

fliF_2	Flagellar M-ring protein	2	4168	7	1646	1646	AJPELHOP_03324	IJBEAHBO_03118		
fliG_1	Flagellar motor switch protein FliG	2	4167	7	1010	1010	AJPELHOP_03323	IJBEAHBO_03117		
fliI_2	Flagellum-specific ATP synthase	2	4165	7	1337	1337	AJPELHOP_03321	IJBEAHBO_03115		
fliP_1	Flagellar biosynthetic protein FliP	2	4173	7	752	752	AJPELHOP_03329	IJBEAHBO_03123		
fliR_1	Flagellar biosynthetic protein FliR	2	4175	7	782	782	AJPELHOP_03331	IJBEAHBO_03125		
fliS_1	Flagellar secretion chaperone FliS	2	4151	7	392	392	AJPELHOP_03298	IJBEAHBO_03092		
flu_1	Antigen 43	2	506	1	3074	3119	AJPELHOP_00724	IJBEAHBO_05277		
focC_1	Chaperone protein FocC	2	1573	100	731	731	AJPELHOP_03132	IJBEAHBO_02298		
folK	2-amino-4-hydroxy-6-hydroxymethyldihydropteridine pyrophosphokinas	2	3367	47	479	479	AJPELHOP_02460	IJBEAHBO_02526		
fyuA	Pesticin receptor	2	2447	28	2021	2021	AJPELHOP_03464	IJBEAHBO_03578		
gamA	putative glucosamine-6-phosphate deaminase 2	2	1600	49	722	722	AJPELHOP_03145	IJBEAHBO_02311		
gatD_1	Galactitol 1-phosphate 5-dehydrogenase	2	2614	315	266	266	AJPELHOP_02288	IJBEAHBO_01519		
gatY_2	D-tagatose-1,6-bisphosphate aldolase subunit GatY	2	1729	57	854	854	AJPELHOP_00264	IJBEAHBO_01927		
glpG_2	Rhomboid protease GlpG	2	4429	378	1877	1877	AJPELHOP_01651	IJBEAHBO_01645		
group_143	Soluble cytochrome b562	2	784	134	302	302	AJPELHOP_04753	IJBEAHBO_04883		
group_1480	Ubiquinone biosynthesis accessory factor UbiK	2	1969	66	290	290	AJPELHOP_00047	IJBEAHBO_00796		
group_1491	Inner membrane protein YhaI	2	1904	392	356	356	AJPELHOP_00114	IJBEAHBO_00729		
group_1498	Tagatose kinase	2	1733	57	962	962	AJPELHOP_00260	IJBEAHBO_01931		
group_1499	Ribose import permease protein RbsC	2	1732	57	989	989	AJPELHOP_00261	IJBEAHBO_01930		
group_1503	Glucitol operon repressor	2	1756	57	815	815	AJPELHOP_00265	IJBEAHBO_01926		
group_1504	Tagatose kinase	2	1757	57	956	956	AJPELHOP_00266	IJBEAHBO_01925		
group_1507	Cytoskeleton bundling-enhancing antitoxin CbeA	2	528	1	407	407	AJPELHOP_00282	IJBEAHBO_00223		
group_1557	Anti-adapter protein IraD	2	718	374	392	392	AJPELHOP_00690	IJBEAHBO_00650		
group_1590	Hydroxypyruvate reductase	2	3	82	947	947	AJPELHOP_00791	IJBEAHBO_00295		
group_1608	putative HTH-type transcriptional regulator	2	4089	8	359	359	AJPELHOP_00961	IJBEAHBO_05356		
group_1620	Protein DedA	2	2	349	659	659	AJPELHOP_01064	IJBEAHBO_01093		
group_1627	putative fimbrial-like protein YfcV	2	5601	62	563	563	AJPELHOP_01085	IJBEAHBO_01114		
group_1644	putative protein	2	5573	1	1412	1412	AJPELHOP_01111	IJBEAHBO_01140		
group_1652	Serine/threonine-protein phosphatase 1	2	5492	1	665	665	AJPELHOP_01120	IJBEAHBO_01149		
group_1702	Very short patch repair protein	2	2462	313	470	470	AJPELHOP_01560	IJBEAHBO_03823		
group_1710	Solute-binding protein	2	94	132	986	986	AJPELHOP_01706	IJBEAHBO_01698		
group_1712	PTS system mannose-specific EIIAB component	2	114	59	428	428	AJPELHOP_01726	IJBEAHBO_01718		
group_1720	Tyrosine recombinase XerC	2	206	113	1607	1607	AJPELHOP_01808	IJBEAHBO_01800		
group_1724	putative lipoprotein YbjP	2	94	348	515	515	AJPELHOP_01856	IJBEAHBO_01848		
group_1744	IS4 family transposase ISCro3	2	3858	135	185	185	AJPELHOP_02012	IJBEAHBO_02125		
group_1766	Undecaprenyl-phosphate 4-deoxy-4-formamido-L-arabinose transferase	2	4274	139	1196	1196	AJPELHOP_02123	IJBEAHBO_02981		
group_1775	Galactose-6-phosphate isomerase subunit LacA	2	435	358	317	317	AJPELHOP_02190	IJBEAHBO_00900		
group_1808	Outer membrane usher protein HtrE	2	3370	47	2600	2600	AJPELHOP_02457	IJBEAHBO_02523		
group_1810	putative fimbrial-like protein YadN	2	3368	47	584	584	AJPELHOP_02459	IJBEAHBO_02525		
group_1875	IS66 family transposase ISEc8	2	2660		278	278	AJPELHOP_02948	IJBEAHBO_03379		
group_1876	putative protein	2	324	319	350	350	AJPELHOP_03012	IJBEAHBO_02441		
group_1884	Mannosyl-D-glycerate transport/metabolism system repressor MngR	2	4600	142	722	722	AJPELHOP_03078	IJBEAHBO_03431		
group_1888	Type-1 fimbrial protein, A chain	2	1572	100	572	572	AJPELHOP_03131	IJBEAHBO_02297		
group_1890	putative outer membrane usher protein ElfC	2	1574	100	2522	2522	AJPELHOP_03133	IJBEAHBO_02299		
group_1981	ISAs1 family transposase ISEc1	2	4177		302	302	AJPELHOP_03344	IJBEAHBO_03138		
group_1982	putative fimbrial subunit LpfE	2	1391	87	524	524	AJPELHOP_03347	IJBEAHBO_03509		
group_1985	putative fimbrial chaperone LpfB	2	1394	87	686	686	AJPELHOP_03350	IJBEAHBO_03512		
group_1986	putative major fimbrial subunit LpfA	2	1395	87	524	524	AJPELHOP_03351	IJBEAHBO_03513		
group_2015	HTH-type transcriptional activator RhaR	2	2453	28	959	959	AJPELHOP_03470	IJBEAHBO_03584		
group_2016	Putative multidrug export ATP-binding/permease protein	2	2455	28	1802	1802	AJPELHOP_03472	IJBEAHBO_03586		
group_2019	Prophage integrase IntA	2	2458	28	749	749	AJPELHOP_03475	IJBEAHBO_03589		
group_2024	Fe(3+) ions import ATP-binding protein FbpC 2	2	2310	389	653	653	AJPELHOP_03573	IJBEAHBO_03735		
group_2060	Protein YoaG	2	2353	321	188	188	AJPELHOP_03875	IJBEAHBO_03964		
group_210	IS200/IS605 family transposase IS609	2	2350		290	290	AJPELHOP_03604	IJBEAHBO_03766		
group_2118	Chain length determinant protein	2	595	330	977	977	AJPELHOP_04273	IJBEAHBO_01458		
group_2138	UDP-3-O-(3-hydroxymyristoyl)glucosamine N-acyltransferase	2	2136	32	956	956	AJPELHOP_04359	IJBEAHBO_02235		
group_2141	Putative dimethyl sulfoxide reductase chain YnfE	2	3778	198	1988	1988	AJPELHOP_04400	IJBEAHBO_04287		
group_2268	IS3 family transposase ISEc16	2	5	23	524	524	AJPELHOP_04713	IJBEAHBO_04864		
group_2270	IS3 family transposase ISEc16	2	3	23	299	299	AJPELHOP_04715	IJBEAHBO_04862		
group_2410	Chaperone protein DnaJ	2	5389	179	752	752	AJPELHOP_05039	IJBEAHBO_05467		
group_2419	IS110 family transposase ISEc20	2	1739	124	767	767	AJPELHOP_05072	IJBEAHBO_05297		
group_2458	IS66 family transposase ISCro1	2	3	141	1571	1571	AJPELHOP_05160	IJBEAHBO_05324		

group_2477	Crossover junction endodeoxyribonuclease RusA	2	33	15	389	389	AJPELHOP_05212	IJBEAHBO_04847		
group_2484	IS66 family transposase ISSfl3	2	1	189	317	317	AJPELHOP_05226	IJBEAHBO_05389		
group_2504	IS110 family transposase IS621	2	1	194	980	980	AJPELHOP_05285	IJBEAHBO_05437		
group_2509	IS66 family transposase ISEc22	2	1	214	1049	1049	AJPELHOP_05294	IJBEAHBO_05454		
group_2517	IS3 family transposase ISEc17	2	3035	119	299	299	AJPELHOP_05320	IJBEAHBO_05480		
group_2519	IS3 family transposase ISKpn37	2	1	174	245	245	AJPELHOP_05335	IJBEAHBO_05499		
group_2543	HTH-type transcriptional regulator PgrR	2	58	180	896	908	AJPELHOP_01891	IJBEAHBO_01883		
group_279	IS3 family transposase IS3	2	3036	119	464	527	AJPELHOP_02325	IJBEAHBO_05481		
group_285	Exodeoxyribonuclease 8	2	2226	42	692	2651	AJPELHOP_04328	IJBEAHBO_02266		
group_301	putative autotransporter	2	26	44	2108	2366	AJPELHOP_03823	IJBEAHBO_03998		
group_416	Cytoskeleton bundling-enhancing antitoxin CbeA	2	703	1	293	368	AJPELHOP_00715	IJBEAHBO_00675		
group_6238	IS3 family transposase IS911	2	21	10	305	305	AJPELHOP_04586	IJBEAHBO_04756		
group_66	Outer membrane protein IcsA autotransporter	2	58	72	854	854	AJPELHOP_03757	IJBEAHBO_04041		
group_6809	ISAs1 family transposase ISEc1	2	1	105	179	179	AJPELHOP_05227	IJBEAHBO_05393		
group_930	Prophage tail fiber assembly protein TfaE	2	4975	17	416	578	AJPELHOP_01504	IJBEAHBO_03767		
hcaR_2	Hca operon transcriptional activator HcaR	2	5348	115	884	884	AJPELHOP_00325	IJBEAHBO_00180		
hcpA	Major exported protein	2	4212	13	482	482	AJPELHOP_02793	IJBEAHBO_04586		
higA-2	Antitoxin HigA-2	2	1005	101	290	290	AJPELHOP_04027	IJBEAHBO_04242		
higB-2	Toxin HigB-2	2	1004	101	311	311	AJPELHOP_04028	IJBEAHBO_04241		
hisC_3	Histidinol-phosphate aminotransferase	2	71	400	821	821	AJPELHOP_04524	IJBEAHBO_04631		
hokE_2	Toxic protein HokE	2	4458	120	152	152	AJPELHOP_01575	IJBEAHBO_01569		
hpcD	5-carboxymethyl-2-hydroxymuconate Delta-isomerase	2	3600	27	380	380	AJPELHOP_00658	IJBEAHBO_00618		
intA_1	Prophage integrase IntA	2	3978	4	1241	1241	AJPELHOP_02879	IJBEAHBO_03310		
intA_4	Prophage integrase IntA	2	487	146	1262	1262	AJPELHOP_04503	IJBEAHBO_04603		
intA_5	Prophage integrase IntA	2	4	15	1220	1229	AJPELHOP_04771	IJBEAHBO_05038		
intQ_5	Putative defective protein IntQ	2	6	15	995	1175	AJPELHOP_04769	IJBEAHBO_05036		
intS_2	Prophage integrase IntS	2	3016	4	1217	1217	AJPELHOP_01276	IJBEAHBO_01293		
intS_3	Prophage integrase IntS	2	1517	26	1169	1169	AJPELHOP_02710	IJBEAHBO_03234		
intS_5	Prophage integrase IntS	2	8	23	665	665	AJPELHOP_04710	IJBEAHBO_04867		
intS_6	Prophage integrase IntS	2	7	23	485	485	AJPELHOP_04711	IJBEAHBO_04866		
intS_7	Prophage integrase IntS	2	2	189	752	752	AJPELHOP_05225	IJBEAHBO_05388		
kdsD_1	Arabinose 5-phosphate isomerase KdsD	2	2	82	596	596	AJPELHOP_00790	IJBEAHBO_00294		
klcA_1	Antirestriction protein KlcA	2	512	1	485	485	AJPELHOP_00719	IJBEAHBO_01430		
lafS	RNA polymerase sigma factor for flagellar operon	2	4155	7	716	716	AJPELHOP_03294	IJBEAHBO_03088		
lafT	Chemotaxis protein LafT	2	4156	7	863	863	AJPELHOP_03293	IJBEAHBO_03087		
ldrD_2	Small toxic polypeptide LdrD	2	1380	86	107	107	AJPELHOP_03976	IJBEAHBO_03623		
ldrD_3	Small toxic polypeptide LdrD	2	1381	86	107	107	AJPELHOP_03977	IJBEAHBO_03624		
ldrD_4	Small toxic polypeptide LdrD	2	1383	86	107	107	AJPELHOP_03979	IJBEAHBO_03626		
levE_2	PTS system fructose-specific EIIB component	2	115	59	485	485	AJPELHOP_01727	IJBEAHBO_01719		
lexA_2	putative HTH-type transcriptional regulator	2	4395	1	689	755	AJPELHOP_02853	IJBEAHBO_03664		
lexA_3	LexA repressor	2	19	15	674	674	AJPELHOP_05203	IJBEAHBO_04838		
lexA_4	LexA repressor	2	32	15	326	326	AJPELHOP_05213	IJBEAHBO_04846		
lexA_6	LexA repressor	2	4101	8	704	704	AJPELHOP_04960	IJBEAHBO_05214		
licT	Transcription antiterminator LicT	2	288	82	824	824	AJPELHOP_00787	IJBEAHBO_00291		
lpfD	putative minor fimbrial subunit LpfD	2	1392	87	1055	1055	AJPELHOP_03348	IJBEAHBO_03510		
lpxG	UDP-2,3-diacylglucosamine pyrophosphatase LpxG	2	3345	335	812	812	AJPELHOP_02482	IJBEAHBO_02548		
ltnD	L-threonate dehydrogenase	2	313	68	908	908	AJPELHOP_00878	IJBEAHBO_00384		
lutR	HTH-type transcriptional regulator LutR	2	1539	71	689	689	AJPELHOP_03871	IJBEAHBO_02333		
lyx	L-xylulose/3-keto-L-gulonate kinase	2	1436	43	1496	1496	AJPELHOP_03385	IJBEAHBO_03547		
malX_1	PTS system maltose-specific EIICB component	2	287	82	1580	1580	AJPELHOP_00788	IJBEAHBO_00292		
malY_1	Protein MalY	2	1	82	1208	1208	AJPELHOP_00789	IJBEAHBO_00293		
manZ_3	PTS system mannose-specific EIID component	2	117	59	857	857	AJPELHOP_01729	IJBEAHBO_01721		
mbeC_1	Mobilization protein MbeC	2	2	85	347	347	AJPELHOP_04894	IJBEAHBO_05349		
mbtI	Salicylate synthase	2	2457	28	1304	1304	AJPELHOP_03474	IJBEAHBO_03588		
mngA	PTS system 2-O-alpha-mannosyl-D-glycerate-specific EIIABC component	2	4601	142	1916	1916	AJPELHOP_03077	IJBEAHBO_03430		
mngB	Mannosylglycerate hydrolase	2	4602	142	2633	2633	AJPELHOP_03076	IJBEAHBO_03429		
mngR_3	Mannosyl-D-glycerate transport/metabolism system repressor MngR	2	67	60	698	698	AJPELHOP_03787	IJBEAHBO_04011		
mshB	1D-myo-inositol 2-acetamido-2-deoxy-alpha-D-glucopyranoside deacetyl	2	3039	139	671	671	AJPELHOP_02124	IJBEAHBO_02980		
msmX_1	Oligosaccharides import ATP-binding protein MsmX	2	2138	32	1127	1127	AJPELHOP_01414	IJBEAHBO_02233		
ndhK	NAD(P)H-quinone oxidoreductase subunit K, chloroplastic	2	5270	376	521	521	AJPELHOP_00403	IJBEAHBO_00101		
ngcG	Diacetylchitobiose uptake system permease protein NgcG	2	2196	32	833	833	AJPELHOP_04355	IJBEAHBO_02239		
nlhH	Carboxylesterase NlhH	2	1003	101	908	908	AJPELHOP_04029	IJBEAHBO_04240		

nohA_3	Prophage DNA-packing protein NohA	2	1	217	224	224	AJPELHOP_05337	IJBEAHBO_05501		
nplT	Neopullulanase	2	2197	32	1799	1799	AJPELHOP_04354	IJBEAHBO_02240		
ompD_1	Outer membrane porin protein OmpD	2	5549	1	1082	1082	AJPELHOP_02868	IJBEAHBO_03649		
ompL	Porin OmpL	2	972	29	692	692	AJPELHOP_04043	IJBEAHBO_04226		
orgA	Oxygen-regulated invasion protein OrgA	2	240	5	581	581	AJPELHOP_00742	IJBEAHBO_00246		
otnC	3-oxo-tetronate 4-phosphate decarboxylase	2	311	68	638	638	AJPELHOP_00876	IJBEAHBO_00382		
otnI	2-oxo-tetronate isomerase	2	310	68	776	776	AJPELHOP_00875	IJBEAHBO_00381		
otnK	3-oxo-tetronate kinase	2	312	68	1262	1262	AJPELHOP_00877	IJBEAHBO_00383		
paaG	1,2-epoxyphenylacetyl-CoA isomerase	2	19	16	788	788	AJPELHOP_03258	IJBEAHBO_03476		
paaH	3-hydroxyadipyl-CoA dehydrogenase	2	16	16	1382	1382	AJPELHOP_03257	IJBEAHBO_03477		
paaI	Acyl-coenzyme A thioesterase PaaI	2	17	16	422	422	AJPELHOP_03256	IJBEAHBO_03478		
paaJ	3-oxoadipyl-CoA/3-oxo-5,6-dehydrosuberyl-CoA thiolase	2	18	16	1205	1205	AJPELHOP_03255	IJBEAHBO_03479		
papC_1	Outer membrane usher protein PapC	2	1967	66	2504	2504	AJPELHOP_00049	IJBEAHBO_00794		
papC_2	Outer membrane usher protein PapC	2	5602	62	2645	2645	AJPELHOP_01084	IJBEAHBO_01113		
papC_3	Outer membrane usher protein PapC	2	4586	95	2450	2450	AJPELHOP_03091	IJBEAHBO_03443		
papD_1	Chaperone protein PapD	2	1966	66	749	749	AJPELHOP_00050	IJBEAHBO_00793		
papD_2	Chaperone protein PapD	2	4585	95	731	731	AJPELHOP_03092	IJBEAHBO_03444		
parD1	Antitoxin ParD1	2	2608	187	251	251	AJPELHOP_02282	IJBEAHBO_01513		
parE1	Toxin ParE1	2	2609	187	296	296	AJPELHOP_02283	IJBEAHBO_01514		
php_2	Phosphotriesterase homology protein	2	4	121	698	698	AJPELHOP_05061	IJBEAHBO_05175		
pikAV	Thioesterase PikA5	2	2449	28	803	803	AJPELHOP_03466	IJBEAHBO_03580		
prtR	HTH-type transcriptional regulator PrtR	2	11	24	569	569	AJPELHOP_04833	IJBEAHBO_05011		
psuK_2	Pseudouridine kinase	2	4018	299	1088	1088	AJPELHOP_02910	IJBEAHBO_03341		
rbsA_1	Ribose import ATP-binding protein RbsA	2	1731	57	1499	1499	AJPELHOP_00262	IJBEAHBO_01929		
rbsB_1	Ribose import binding protein RbsB	2	1730	57	890	890	AJPELHOP_00263	IJBEAHBO_01928		
rcbA_1	Double-strand break reduction protein	2	2228	42	188	188	AJPELHOP_04330	IJBEAHBO_02264		
recT_1	Protein RecT	2	2227	42	809	809	AJPELHOP_04329	IJBEAHBO_02265		
rfbB	dTDP-glucose 4,6-dehydratase	2	580	46	1076	1085	AJPELHOP_02241	IJBEAHBO_01472		
rhsC	Protein RhsC	2	912	77	737	2006	AJPELHOP_02778	IJBEAHBO_03303		
rhsD_3	Protein RhsD	2	4	105	491	491	AJPELHOP_05230	IJBEAHBO_05390		
rop_2	Regulatory protein rop	2	8	85	191	191	AJPELHOP_04888	IJBEAHBO_05348		
rrrD_4	Lysozyme RrrD	2	2	74	497	497	AJPELHOP_05088	IJBEAHBO_05231		
rspB	Starvation-sensing protein RspB	2	3636	96	1019	1019	AJPELHOP_04407	IJBEAHBO_04280		
rsxC	Ion-translocating oxidoreductase complex subunit C	2	2987	284	2222	2222	AJPELHOP_01305	IJBEAHBO_01322		
rusA_6	Crossover junction endodeoxyribonuclease RusA	2	6	104	362	362	AJPELHOP_05045	IJBEAHBO_05260		
sasA	Adaptive-response sensory-kinase SasA	2	1	15	1718	1718	AJPELHOP_04774	IJBEAHBO_05041		
setA	Sugar efflux transporter A	2	3450	195	1160	1160	AJPELHOP_00531	IJBEAHBO_00493		
setC	Sugar efflux transporter C	2	1531	26	1184	1184	AJPELHOP_03834	IJBEAHBO_02371		
sgbE	L-ribulose-5-phosphate 4-epimerase SgbE	2	1439	43	695	695	AJPELHOP_03388	IJBEAHBO_03550		
sgbH	3-keto-L-gulonate-6-phosphate decarboxylase SgbH	2	1437	43	662	662	AJPELHOP_03386	IJBEAHBO_03548		
sgrT	Putative inhibitor of glucose uptake transporter SgrT	2	3451	195	131	131	AJPELHOP_00532	IJBEAHBO_00494		
siaM	Sialic acid TRAP transporter large permease protein SiaM	2	1434	43	1277	1277	AJPELHOP_03383	IJBEAHBO_03545		
sopA	E3 ubiquitin-protein ligase SopA	2	2840	130	1328	1328	AJPELHOP_03206	IJBEAHBO_00950		
sorM_2	PTS system sorbose-specific EIID component	2	2844	300	458	458	AJPELHOP_03178	IJBEAHBO_00978		
spaS_2	Surface presentation of antigens protein SpaS	2	233	5	362	362	AJPELHOP_00735	IJBEAHBO_00239		
srlR_3	Glucitol operon repressor	2	314	68	767	767	AJPELHOP_00879	IJBEAHBO_00385		
symE	Toxic protein SymE	2	3578	41	341	341	AJPELHOP_00673	IJBEAHBO_00633		
tagD	Glycerol-3-phosphate cytidylyltransferase	2	4163	7	395	395	AJPELHOP_03319	IJBEAHBO_03113		
tir	Translocated intimin receptor Tir	2	2121	73	1616	1616	AJPELHOP_03934	IJBEAHBO_04659		
torI	Response regulator inhibitor for tor operon	2	9	23	200	200	AJPELHOP_04709	IJBEAHBO_04868		
ulaE_2	L-ribulose-5-phosphate 3-epimerase UlaE	2	1438	43	860	860	AJPELHOP_03387	IJBEAHBO_03549		
uvrB_1	UvrABC system protein B	2	1518	26	1859	1859	AJPELHOP_02709	IJBEAHBO_03233		
vapB_1	Antitoxin VapB	2	5154	206	227	227	AJPELHOP_00388	IJBEAHBO_00116		
vapC_1	tRNA(fMet)-specific endonuclease VapC	2	5155	206	398	398	AJPELHOP_00387	IJBEAHBO_00117		
vnfA	Nitrogen fixation protein VnfA	2	4170	7	986	986	AJPELHOP_03326	IJBEAHBO_03120		
xapA	Purine nucleoside phosphorylase 2	2	5346	115	833	833	AJPELHOP_00327	IJBEAHBO_00178		
xapB	Xanthosine permease	2	5347	115	1256	1256	AJPELHOP_00326	IJBEAHBO_00179		
xerC_1	Tyrosine recombinase XerC	2	4801	67	1070	1070	AJPELHOP_00979	IJBEAHBO_01007		
xerC_6	Tyrosine recombinase XerC	2	4257	30	1277	1277	AJPELHOP_02046	IJBEAHBO_03058		
yadC_1	putative fimbrial-like protein YadC	2	3375	47	455	455	AJPELHOP_02452	IJBEAHBO_02518		
yadC_2	putative fimbrial-like protein YadC	2	3374	47	542	542	AJPELHOP_02453	IJBEAHBO_02519		
yadK	putative fimbrial-like protein YadK	2	3373	47	596	596	AJPELHOP_02454	IJBEAHBO_02520		

yadL	putative fimbrial-like protein YadL	2	3372	47	602	602	AJPELHOP_02455	IJBEAHBO_02521		
yadM	putative fimbrial-like protein YadM	2	3371	47	569	569	AJPELHOP_02456	IJBEAHBO_02522		
yadV	putative fimbrial chaperone YadV	2	3369	47	740	740	AJPELHOP_02458	IJBEAHBO_02524		
yafP	putative N-acetyltransferase YafP	2	4243	370	452	452	AJPELHOP_03290	IJBEAHBO_03084		
ybbA	putative protein YbbA	2	2199	32	839	839	AJPELHOP_04352	IJBEAHBO_02242		
ybcO_2	Putative nuclease YbcO	2	5	104	290	290	AJPELHOP_05046	IJBEAHBO_05261		
ybgD_1	putative fimbrial-like protein YbgD	2	1968	66	551	551	AJPELHOP_00048	IJBEAHBO_00795		
ybgD_2	putative fimbrial-like protein YbgD	2	4588	95	173	566	AJPELHOP_03089	IJBEAHBO_03442		
ybiA	N-glycosidase YbiA	2	165	394	449	449	AJPELHOP_03624	IJBEAHBO_04393		
ycfZ	Inner membrane protein YcfZ	2	3721	211	788	788	AJPELHOP_02512	IJBEAHBO_03146		
ydiF_2	Acetate CoA-transferase YdiF	2	2244	168	701	701	AJPELHOP_01373	IJBEAHBO_01390		
yehR_1	putative lipoprotein YehR	2	2658	114	131	131	AJPELHOP_02322	IJBEAHBO_01554		
yfcO	putative protein YfcO	2	5607	62	851	851	AJPELHOP_01079	IJBEAHBO_01108		
yfcP	putative fimbrial-like protein YfcP	2	5606	62	539	539	AJPELHOP_01080	IJBEAHBO_01109		
yfcQ	putative fimbrial-like protein YfcQ	2	5605	62	488	488	AJPELHOP_01081	IJBEAHBO_01110		
yfcR	putative fimbrial-like protein YfcR	2	5604	62	509	509	AJPELHOP_01082	IJBEAHBO_01111		
yfcS	putative fimbrial chaperone YfcS	2	5603	62	752	752	AJPELHOP_01083	IJBEAHBO_01112		
yfgJ	putative protein YfgJ	2	5244	310	215	215	AJPELHOP_00425	IJBEAHBO_00079		
ygaV_2	putative HTH-type transcriptional regulator YgaV	2	1330	110	296	296	AJPELHOP_04005	IJBEAHBO_04109		
ygbN	Inner membrane permease YgbN	2	309	68	1364	1364	AJPELHOP_00874	IJBEAHBO_00380		
ygcB	CRISPR-associated endonuclease/helicase Cas3	2	438	31	1517	2699	AJPELHOP_00849	IJBEAHBO_00354		
ygeK	putative response regulatory protein YgeK	2	243	5	443	443	AJPELHOP_00745	IJBEAHBO_00249		
ygfF	putative oxidoreductase YgfF	2	165	325	743	743	AJPELHOP_01769	IJBEAHBO_01761		
ygiZ	Inner membrane protein YgiZ	2	1989	288	332	332	AJPELHOP_00032	IJBEAHBO_00811		
ygjK_1	Glucosidase YgjK	2	1717	395	920	920	AJPELHOP_00086	IJBEAHBO_00757		
yhcG_1	Putative nuclease YhcG	2	1728	345	548	548	AJPELHOP_00223	IJBEAHBO_01968		
yhcH_2	putative protein YhcH	2	1432	43	467	467	AJPELHOP_03381	IJBEAHBO_03543		
yiaJ	DNA-binding transcriptional repressor YiaJ	2	1430	43	848	848	AJPELHOP_03379	IJBEAHBO_03541		
yiaM	2,3-diketo-L-gulonate TRAP transporter small permease protein YiaM	2	1433	43	473	473	AJPELHOP_03382	IJBEAHBO_03544		
yiaO	2,3-diketo-L-gulonate-binding periplasmic protein YiaO	2	1435	43	986	986	AJPELHOP_03384	IJBEAHBO_03546		
yibA_1	Protein YibA	2	913	77	842	842	AJPELHOP_02777	IJBEAHBO_03302		
yibA_2	Protein YibA	2	2	105	245	245	AJPELHOP_05228	IJBEAHBO_05392		
yieH	6-phosphogluconate phosphatase	2	1603	49	665	665	AJPELHOP_03148	IJBEAHBO_02314		
yjeO	Inner membrane protein YjeO	2	482	201	302	302	AJPELHOP_03502	IJBEAHBO_02958		
yjgN_1	Inner membrane protein YjgN	2	762	369	740	740	AJPELHOP_04494	IJBEAHBO_04612		
yjiK_1	putative protein YjiK	2	3562	48	608	608	AJPELHOP_00683	IJBEAHBO_00643		
yjiK_2	putative protein YjiK	2	1680	127	818	818	AJPELHOP_02143	IJBEAHBO_00853		
ylcG	putative protein YlcG	2	5551	1	137	137	AJPELHOP_02866	IJBEAHBO_03651		
ylpA_3	Lipoprotein YlpA	2	2038	11	254	254	AJPELHOP_04412	IJBEAHBO_04526		
ymfA	Inner membrane protein YmfA	2	3722	211	461	461	AJPELHOP_02511	IJBEAHBO_03145		
ymiC	Protein YmiC	2	281	256	95	95	AJPELHOP_04299	IJBEAHBO_02456		
yqcG	putative protein YqcG	2	462	202	140	140	AJPELHOP_00831	IJBEAHBO_00335		
yqiK_1	Inner membrane protein YqiK	2	1716	291	851	851	AJPELHOP_00055	IJBEAHBO_00788		
